# Annual Meeting of the International Society of Cancer Metabolism (ISCaM): Cancer Metabolism

**DOI:** 10.3389/fonc.2018.00329

**Published:** 2018-10-03

**Authors:** Sofia Avnet, Nicola Baldini, Lucie Brisson, Angelo De Milito, Angela M. Otto, Silvia Pastoreková, Paolo E. Porporato, Gyorgy Szabadkai, Pierre Sonveaux

**Affiliations:** ^1^Orthopaedic Pathophysiology and Regenerative Medicine Unit, Istituto Ortopedico Rizzoli IRCCS, Bologna, Italy; ^2^Department of Biomedical and Neuromotor Sciences, University of Bologna, Bologna, Italy; ^3^INSERM UMR1069, Nutrition, Croissance et Cancer, Université François-Rabelais, Tours, France; ^4^Department of Oncology-Pathology, Karolinska Institute, and Sprint Bioscience, Stockholm, Sweden; ^5^Munich School of BioEngineering, Technical University of Munich, Munich, Germany; ^6^Biomedical Research Center, Slovak Academy of Sciences, Bratislava, Slovakia; ^7^Department of Molecular Biotechnology and Health Science, Molecular Biotechnology Center, University of Torino, Torino, Italy; ^8^Department of Biomedical Sciences, University of Padua, Padua, Italy; ^9^Department of Cell and Developmental Biology, Consortium for Mitochondrial Research, University College London, London, United Kingdom; ^10^The Francis Crick Institute, London, United Kingdom; ^11^Pole of Pharmacology, Institut de Recherche Expérimentale et Clinique, Université catholique de Louvain, Brussels, Belgium

**Keywords:** tumor metabolism, proton dynamics, tumor microenvironment, cancer imaging, cancer therapy

## Abstract

Tumors are metabolic entities wherein cancer cells adapt their metabolism to their oncogenic agenda and microenvironmental influences. Metabolically different cancer cell subpopulations collaborate to optimize nutrient delivery with respect to immediate bioenergetic and biosynthetic needs. They can also metabolically exploit host cells. These metabolic networks are directly linked with cancer progression, treatment, resistance, and relapse. Conversely, metabolic alterations in cancer are exploited for anticancer therapy, imaging, and stratification for personalized treatments. These topics were addressed at the 4th annual meeting of the International Society of Cancer Metabolism (ISCaM) in Bertinoro, Italy, on 19–21 October 2017.

## Introduction

Tumors are metabolic entities wherein cancer cells adapt their metabolism to their oncogenic agenda and microenvironment. This highly adaptive metabolic behavior is fundamental for the different crucial steps of cancer progression, including the ability to sustain energy-consuming cell proliferation, migration at distant sites and in different tissues with respect to the tissue of origin, long-term survival in a quiescent state in hostile conditions and drug resistance. To address these important aspects of tumor biology, the International Society of Cancer Metabolism (ISCaM) was founded in 2014 as a logical expansion of the International Society for the Study of Proton Dynamics in Cancer (ISPDC) that operated from 2009 to 2014. ISCaM's scope is to improve communication and to foster collaboration between scientists engaged in acidity, proton dynamics and all aspects of metabolism in tumors and their microenvironment. On 19–21 October 2017, ISCaM held its 4th annual meeting in Bertinoro, Italy (1–3). This 3-day event covered several aspects of cancer metabolism, including interactions between cancer and host cells, microenvironmental characteristics such as local hypoxia and acidosis, and subcellular functions, including autophagy, lysosome and mitochondrial deregulation and lipid metabolism. Experts and trainees in genomics, molecular biology, biochemistry, cell biology, imaging, diagnostics, computational analysis, and nutrition exchanged their experiences and ideas to foster the development of effective treatments based on a comprehensive understanding of cancer. In this respect, during the opening lecture, Ravid Straussmann described how bacteria in the tumor microenvironment affect chemosensitivity. He elegantly demonstrated that tumors are highly complex ecosystems that include multiple components of the tumor microenvironment and a tumor microbiome. The cancer microenvironment and its connection with cancer metabolism were at the core of ISCaM2017 and were addressed in 10 thematic oral sessions that were accompanied by solid informative poster sessions.

## Microenvironment and cancer metabolism

Cancer metabolism generates acidic products that reach the stroma. Azbeta Hulikova explained that stromal myofibroblasts absorb protons via anion exchanger 2 and distribute them across the stroma through gap junctions. Stromal cells thereby optimize acid distribution in tumors, which facilitates tumor growth. Exchanges also concern different types of cancer cells. Indeed, Marie-Luce Vignais showed that mesenchymal cells establish nanotube connections to transfer mitochondria to cancer stem cells that gain enhanced oxidative phosphorylation, proliferation and invasiveness. Next talks focused on stromal cells. Silvia Lemma first explained that lactate produced by monocytes and macrophages fuels the osteolytic activity of osteoclast-like giant cells of the bone marrow. These cells take up lactate via monocarboxylate transporter 1 (MCT1) and use it as a source of energy for bone degradation. Lactate can also fuel pyruvate anaplerosis, as shown by Maria Gkiouli in breast cancer. Focusing on cancer-associated fibroblasts (CAFs), Serena Martinelli reported increased glucose and lactate uptake by succinate dehydrogenase (SDH)-deficient pheochromocytoma-derived cancer cells when co-cultured with CAFs. Cancer cells gained increased migratory capabilities and shifted from collective to individual migration in 3D spheroid co-cultures. CAFs further induced a lactate-dependent activation of the sirtuin1/peroxisome proliferator-activated receptor γ coactivator-1α (SIRT1/PGC1α) axis to boost mitochondrial metabolism in prostate carcinoma cells, as evidenced by Luigi Ippolito. In macrophages, Alessandra Castegna reported that glutamine synthetase represents a potential antimetastatic target as its inhibition promotes an antimetastatic M2 to M1 shift and activates transcritption factor hypoxia-inducible factor-1 (HIF-1). Together, these talks illustrated the metabolic contribution of the tumor microenvironment to cancer progression.

## Metabolic control of cancer stemness

Cancer stem cells have important roles in cancer initiation, progression, and drug resistance. Speakers discussed the crosstalk between cancer stem cells and the tumor microenvironment, including hypoxia, local acidosis, and metabolic substrate availability. Margherita Cortini first described a new cancer stem cell isolation protocol from osteosarcomas. The team used 3D models with conditioned medium from mesenchymal stem cells to highlight the role of acidic pH and hypoxia on stemness and chemotherapy efficiency. In pancreatic ductal adenocarcinoma (PDAC), EACR-sponsored lecturer Stephan J Reshkin focused on the influence of stromal collagen I on the metabolic phenotype and PDAC stem cells. Tokuhiro Chano then showed that RAB39A, a member of the RAS oncogene family, was selectively expressed in cancer cells and induced by hypoxia. RAB39A and its downstream effector RXRB are associated with cancer stemness and tumorigenesis. Concerning metabolic substrates, Martina Poteti showed that glutamine availability suppressed the expression of oncoprotein BCR/Abl in leukemia stem cells, rendering them resistant to BCR/Abl tyrosine kinase inhibitors. Irene Bertolini focused on microvesicles secreted by neurospheres, which are enriched in glioma stem cells and increase the growth of recipient glioblastoma cells. pH regulation through vacuolar (V)-ATPase was identified to control microvesicle content. Conversely, cancer cell metabolism can influence cancer stemness, as illustrated by Alessandro Carrer who reported that acetyl-CoA production by ATP-citrate lyase and subsequent histone acetylation are early events in the tumorigenesis of Kras-driven PDAC.

## pH dynamics and carbonic anhydrase IX (CA9) targeting

pH regulation is at the core of tumor metabolism. One important component of pH regulation is carbonic anhydrase IX (CA9) that exists both as a transmembrane protein and as a shed ectodomain circulating in the blood of tumor patients. Silvia Pastorekova showed that CA9 ectodomain cleavage affects the cancer cell phenotype and microenvironment. Shonagh Russell then demonstrated that CA9 overexpression supports the glycolytic phenotype of cancer cells and increases their metastatic propensity. Holger M Becker further explained that CA9 increases the transport activity of MCT1 and MCT4 through direct interaction, thus facilitating lactate efflux from hypoxic cancer cells and extracellular acidosis. In melanoma, Ines Boehme showed that extracellular acidosis triggers a senescence-like phenotype associated with cell reprogramming. Gemma Di Pompo further described that acidity promotes osteolysis, and she suggested anti-acid approaches to reduce osteolytic cancer lesions. Addressing additional pH regulatory systems, Agustin Asuaje reported that voltage-gated proton channel Hv1 is essential for pH homeostasis and the survival of leukemic Jurkat T cells. He proposed Hv1 as a target to induce intracellular acidification. Jessica Iorio further illustrated that inhibitors of ion channels and transporters modulate intracellular pH in colorectal cancer cells in an extracellular matrix-dependent manner that involved the β1-integrin/potassium channel Kv11.1 complex. At the clinical level, Katsuyuki Kusuzaki presented a promising trial using acridine orange to sensitize patients with malignant musculoskeletal tumors to radiotherapy.

## Autophagy and lysosomes

Mila Gugnoni reported that cadherin-6 (CDH6) promotes the metastatic progression of thyroid cancer. Loss of CDH6 disrupted the cellular architecture and attenuated epithelial-to-mesenchymal transition (EMT) features of thyroid cancer cells. CDH6 reduced mitophagy, and mitochondrial fixation promoted cytoskeleton remodeling, leading to collective cell migration. A high expression of CDH6 was associated with a poor prognosis in papillary thyroid carcinoma patients. Yasmin Yu then reported the activities of a novel, selective phosphatidylinositol-3-kinase Vps34 inhibitor developed by Sprint Bioscience, which inhibits autophagy and decreases the growth of human breast carcinomas. Inhibition of autophagy by Vps34 inhibitors increased sunitinib sensitivity of breast cancer cells grown as monolayers and multicellular spheroids. Together with recent evidence of Vps34-mediated regulation of cellular metabolism, the new data made this lipid kinase a new interesting target in oncology and metabolic diseases. Miriam Martini identified another lipid kinase, PIK3-C2γ, as a PDAC tumor suppressor. Deletion of the *PIK3C2G* gene in the KPC pancreatic cancer mouse model initiated and promoted tumor development, explaining why low PI3K-C2γ expression is associated with poor survival and chemoresistance. Pharmacological inhibition of autophagy in *PIK3C2G*^−/−^ KPC tumors led to tumor regression, suggesting that the metabolic phenotype of PI3K-C2γ-deficient tumors can be exploited therapeutically.

## Lipid metabolism

The increasingly recognized importance of lipids in cancer has recently prompted rapid advances in lipidomics and related technologies, including mass spectrometry (MS), liquid chromatography tandem-mass spectrometry (LC-MS/MS) and nuclear magnetic resonance (NMR) spectroscopy. In response to stress conditions, cancer cells display strong lipid avidity and a shift to lipid metabolism through hyperactivation of lipogenic enzymes, enhancement of *de novo* lipid synthesis, increased uptake of exogenous lipids, accumulation of lipid droplets, and increased lipolysis. Stefania Raimondo showed that intracellular phospholipase A1 family member DDHD1 supports colon cancer cell proliferation and survival by modulating PI3K/Akt and ERK signaling pathways, which can be repressed with siRNAs against DDHD1. This new target was reported to be cancer-specific. Anti-angiogenic therapies have a stressing impact on cancers and may activate lipid metabolism as a mechanism of resistance to hypoxia. Using LC-MS analysis, Carolina Venturoli showed that anti-VEGF therapy enhances the total level of fatty acids and the intracellular concentration of diacylglycerols and triacylglycerols in xenograft models of ovarian cancer. However, no changes were observed in the expression of enzymes involved in fatty acid synthesis and uptake. Transcriptome analyses rather revealed increased expression of phospholipase A2, a key enzyme for the release of fatty acids from glycerol.

## Mitochondria in cancer

Mitochondria play a central role in energy metabolism, biosynthesis, calcium, and redox homeostasis and signaling. Focusing on redox stress, Götz Hartleben found that mTORC1-inhibitor tuberous sclerosis 1 (TSC1) ensures homeostasis between oncogenic factors MYC and mTORC1 through the repression of both mitochondrial oxygen consumption and mitochondrial reactive oxygen species (mtROS) production In Burkitt's lymphoma cells. Gloria Scattolin further reported that drugs increasing mtROS production or decreasing mtROS scavenging induce the death of T-lymphoblastic leukemia cells via activation of mitochondrial protease OMA1. Beyond mtROS, Samantha C Higgins tested drug repositioning to target mitochondrial functions in gliomas. She showed that tricyclic antidepressant clomipramine induces a cell cycle arrest in G2/M phase, inhibits complexes I and III of the electron transport chain (ETC), triggers mitochondrial depolarization, cytochrome c release and apoptosis. However, upon ETC complex I inactivation, Luisa Iommarini highlighted that osteosarcoma cells increased their glycolytic flux, glutamine carboxylation and lipid metabolism for survival. Cancer is also a systemic metabolic disease. Indeed, in cachectic muscles of pancreatic and colorectal cancer patients, Adeline Dolly reported decreased mitochondrial oxygen consumption linked to energy production, which reflected strong mitochondrial alterations in cachectic muscles. Heme is involved in mitochondrial metabolism and is also a strong pro-oxidant. Veronica Fiorito found that plasma membrane-bound heme exporter FLVCR1a controls heme homeostasis in a subpopulation of colorectal cancer cells, which potentially opens new therapeutic perspectives.

## Metabolic control of cancer behavior

A fundamental question in cancer metabolism is how limitations of glucose and glutamine availability affect the metabolic cancer phenotype. In MCF7 breast cancer cells, Angela M Otto found that glucose-derived ^13^C is present in both glycolysis and TCA-cycle intermediates, varying with the concentrations and ratios of glucose and glutamine. Especially at limiting glucose levels, ^13^C profiles pointed at pyruvate carboxylation and pyruvate recycling. On the other hand, overexpression of mitochondrial isocitrate dehydrogenase 2 (IDH2) can perturb the metabolic balance, which made breast cancer cells more sensitive to other metabolic impairments. As Georgina D Barnabas showed, knocking down two enzymes of serine synthesis, phosphoglycerate dehydrogenase and phosphoserine aminotransferase, reduced glycolysis and inhibited cancer cell survival. She proposed new combinations for synthetic lethality. Amedeo Columbano focused on early hepatocarcinomas, in which downregulation of succinate dehydrogenase by tumor necrosis factor receptor-associated protein 1 (TRAP1) during tumor progression increased MCT4 expression and glucose-6-phosphate dehydrogenase (G6PDH) activity. Conversely, silencing transcription factor NRF2 increased the expression of miRNA-1, a G6PDH-tageting miR, thus deregulating the pentose phosphate pathway. Katiuscia Bianchi presented an interesting novel interaction between the innate immune system and serine metabolism in tumors, mediated by direct phosphorylation and transcriptional regulation of enzymes in the serine biosynthesis pathway. Extracellular ATP, which can also be a mediator of tumor inflammation, is detected in tumors where it activates ionotropic receptor P2X7. Lucie Brisson reported that P2X7 overexpression is associated with metastatic features in various cancers, supporting P2X7 antagonists as anticancer drug candidates.

In a second session on the same topic, Elena Piccinin identified that PGC1β drives metabolic rewiring to promote hepatocellular cancer progression. A little appreciated issue in the study of cancer metabolism is the identification of specific metabolic inhibitors. Several widely used molecules (i.e., 2-deoxyglucose and 3-bromopyruvate) are indeed not selective for blocking the glycolytic flux, making the development of novel drugs an impellent need. To fill this gap, Adam Zweifach developed a novel screening strategy to pinpoint molecules specifically blocking glycolysis. Ferdinando Chiaradonna further described the development of inhibitors targeting aberrant protein glycosylation in triple-negative breast cancer. Identification of metabolic targets in cancer has valuable therapeutic potential, as illustrated by Kiran K Velpula when targeting pyruvate dehydrogenase kinase 1 in temozolomide-resistant glioblastoma cells; and Riva Shmulevich who used KLOTHO, a soluble tumor suppressor, as anticancer treatment. Identification of proper antimetabolic therapies also requires analyzing cancer metabolic subtypes. Hence, Michela Menegollo focused on differential carbon source dependencies of breast cancer subtypes. Metabolic deregulation in cancer was also addressed from the systemic standpoint. In particular, Lindsay A Broadfield reported effects of metformin directly on the microbiome, which can alter host metabolism.

## Targeting mitochondria and metabolism

New treatments are currently emerging selectively targeting tumor cell metabolism. Ivana Kurelac presented new data on ETC complex I inhibition, highlighting decreased activation of HIF-1 and reduced angiogenesis in complex I-deficient osteosarcoma and colorectal cancer cells. However, stromal cells partly rescued angiogenesis. Agnese De Mario focused on the mitochondrial calcium uniporter (MCU) that gates mitochondrial calcium uptake. MCU depletion decreased tumor growth and metastasis, whereas increased mitochondrial calcium uptake with two compounds newly identified by De Mario's team had opposite effects. Christos Chinopoulos reported that highly glutaminolytic and metastatic glioblastoma VM-M3 cells rely on a high activity of succinate-coA ligase to produce energy-rich phosphates when cellular respiration is impaired. Knocking-down subunit Suclg1 killed the cells, pointing at succinate-coA ligase as a potential anticancer target. When comparing responses of glioma and breast cancer cells to metabolic modulators 2-deoxyglucose, dichloroacetate, and phenformin, Diana Tavares-Valente identified cell type-specific metabolic changes indicative of different sensitivities to the treatments. Nevertheless, pretreatment with these inhibitors sensitized glioblastoma cells to temozolomide and breast cancer cells to doxorubicin chemotherapy *in vivo*, which should prompt further evaluation of such strategies.

## Imaging of cancer metabolism

This session covered advanced approaches to understand the spatial distribution of metabolites in tumors, answering questions about basic metabolic changes and adaptation to the tumor microenvironment and about inherent metabolic heterogeneity. Valery V Khramtsov summarized recent efforts to use paramagnetic resonance spectroscopy and imaging to characterize oxygen, pH and inorganic phosphate distribution in *in vivo* tumor models. Interestingly, intratumor phosphate content reflected oxygen supply but not pH, suggesting that phosphate accumulation reflects mitochondrial activity, while pH is independent of the hypoxic state of tumors. Annasofia Anemone reported similar findings using breast cancer xenograft models and MRI-based approaches to map tumor hypoxia and acidosis *in vivo*. Her data suggested that hypoxia and acidosis are causally associated, but not spatially correlated. Eleonora Cavallari confirmed the association between lactate dehydrogenase activity and hypoxia in cancer cells using MR analysis of hyperpolarized [^1−13^C]-pyruvate metabolism. Giuseppe Ferrauto followed by compellingly demonstrating that multiparametric MRI is a valuable tool for tumor staging, in particular by providing indirect information about the “cellularity” of prostate tumors, which correlates with tumor invasiveness. He also presented a promising novel approach utilizing the frequency-encoded contrast of chemical exchange saturation transfer agent YB-HPDO3A for glioblastoma pH mapping in collaboration with Hana Lahrech. Mikhail M Dikov closed the session by presenting a novel application of EPR-based phosphate imaging, complementing the first talk in the session. He showed how to use these data to predict immune depletion and the metastatic potential of tumors.

## Cancer metabolism and drug resistance

Tumors can develop metabolic adaptations to cope with the toxicity of chemotherapeutic agents. Giulia Girolimetti showed that accumulation of mitochondrial DNA mutations significantly contributes to resistance to paclitaxel in *in vivo* tumor models by altering mitochondrial activity and consequent PGC1α-driven adaptive mitochondrial biogenesis. Other speakers addressed the question of whether the usage of different nutrients might contribute to the regulation of drug sensitivity. For instance, Sepideh Aminzadeh-Gohari showed that ketogenic diet can increase the tumor response to combination chemotherapy *in vivo*. Novel targets to overcome drug resistance were presented as well. Valentina Audrito showed that nicotinamide phosphoribosyltransferase is a potential target in BRAF-mutated metastatic melanoma, and Zinger Lotem proposed that inhibition of the mTORC1/2-Akt axis can counteract anti-estrogen resistance associated with estrogen receptor-activating mutations in breast cancer cell lines.

## Conclusions

Cancer metabolism is at the core of cancer research, and several therapeutic interventions targeting precise metabolic enzymes, receptors, and transporters are currently reaching clinical evaluation stages. Accordingly, in the closing lecture, Antonio Moschetta concluded the ISCaM2017 meeting by providing a broad overview of the importance of nutrients, genes and metabolism in cancer, largely exemplified with the case of hepatocarcinomas.

ISCaM aims to promote interactions between basic and clinical researchers and between young and senior scientists. At ISCaM2017, those interactions were facilitated by the relatively small size of the meeting (132 participants) that was open to ISCaM members only this year, and by its international composition (attendees represented 20 countries). Social activities and meet-the-expert sessions were organized. Oral and poster communications were dispatched to sessions focusing on emerging aspects of cancer metabolism that were co-chaired by a principal and by a young investigator. Prizes were awarded to young investigators who delivered best scientific presentations. The best oral presentation prize went to Mila Gugnoni for her study highlighted above, and the best poster presentation prize to Alessandra Iscaro for her study on the microenvironmental control of immune modulation during carcinoma progression. Travel grants were also attributed to young scientists based on the scientific merit of submitted abstracts. The 10 awardees were Agustin Asuaje, Valentina Audrito, Georgina Barnabas, Lindsay Broadfield, Alessandro Carrer, Giuseppe Ferrauto, Zinger Lotem, Felipe Muñoz Cordova, Riva Shmulevich, and Shonag Russell. Finally, the ISCaM board was renewed at the General Assembly, and Silvia Pastorekova was appointed President-Elect to ensure the Presidency of ISCaM from the annual meeting ISCaM2018.

Conclusively, ISCaM2017 provided the audience with most recent discoveries on metabolic networks in cancer. This major topic of investigation will continue to be discussed and updated at the ISCaM2018 meeting in Bratislava, Slovakia, 17–20 October 2018 (https://www.iscams.org/meeting/iscam2018-%E2%80%93-5th-annual-meeting-%E2%80%93-metabolic-adaptations-and-targets-cancer-%E2%80%93-bratislava).

## Author contributions

Authors are/were members of the Board of the International Society of Cancer Metabolism (ISCaM). All authors equally contributed to this manuscript.

### Conflict of interest statement

The authors declare that the research was conducted in the absence of any commercial or financial relationships that could be construed as a potential conflict of interest.
